# Important Trends in UCP3 Investigation

**DOI:** 10.3389/fphys.2019.00470

**Published:** 2019-04-30

**Authors:** Elena E. Pohl, Anne Rupprecht, Gabriel Macher, Karolina E. Hilse

**Affiliations:** ^1^Institute of Physiology, Pathophysiology and Biophysics, University of Veterinary Medicine, Vienna, Austria; ^2^Institute of Pharmacology and Toxicology, Rostock University Medical Center, Rostock, Germany

**Keywords:** proton transport, uncoupling protein expression pattern, fatty acid beta-oxidation, cell metabolism, mitochondria

## Abstract

Membrane uncoupling protein 3 (UCP3), a member of the mitochondrial uncoupling protein family, was discovered in 1997. UCP3′s properties, such as its high homology to other mitochondrial carriers, especially to UCP2, its short lifetime and low specificity of UCP3 antibodies, have hindered progress in understanding its biological function and transport mechanism over decades. The abundance of UCP3 is highest in murine brown adipose tissue (BAT, 15.0 pmol/mg protein), compared to heart (2.7 pmol/mg protein) and the gastrocnemius muscle (1.7 pmol/mg protein), but it is still 400-fold lower than the abundance of UCP1, a biomarker for BAT. Investigation of UCP3 reconstituted in planar bilayer membranes revealed that it transports protons only when activated by fatty acids (FA). Although purine nucleotides (PN) inhibit UCP3-mediated transport, the molecular mechanism differs from that of UCP1. It remains a conundrum that two homologous proton-transporting proteins exist within the same tissue. Recently, we proposed that UCP3 abundance directly correlates with the degree of FA β-oxidation in cell metabolism. Further development in this field implies that UCP3 may have dual function in transporting substrates, which have yet to be identified, alongside protons. Evaluation of the literature with respect to UCP3 is a complex task because (i) UCP3 features are often extrapolated from its “twin” UCP2 without additional proof, and (ii) the specificity of antibodies against UCP3 used in studies is rarely evaluated. In this review, we primarily focus on recent findings obtained for UCP3 in biological and biomimetic systems.

## Introduction

The role of brown adipose tissue (BAT) in obesity was suggested 40 years ago based on the ability of brown fat mitochondria to dissipate energy as heat (Parascandola, 1974; Himms-Hagen, 1979; Harper et al., 2001). However, until 2009 BAT was only linked to hibernating mammals and newborns (Heaton, 1972; Spiegelman and Flier, 2001). The discovery of active BAT in human adults and identification of a third type of fat cells – beige or brite adipocytes (brite adipose tissue, BrAT) (Petrovic et al., 2010; Wu et al., 2012) – reintroduced BAT into research focus as a target for the treatment of obesity and other metabolic disorders (Cypess et al., 2009; Virtanen et al., 2009; Chondronikola and Sidossis, 2018).

**FIGURE 1:**
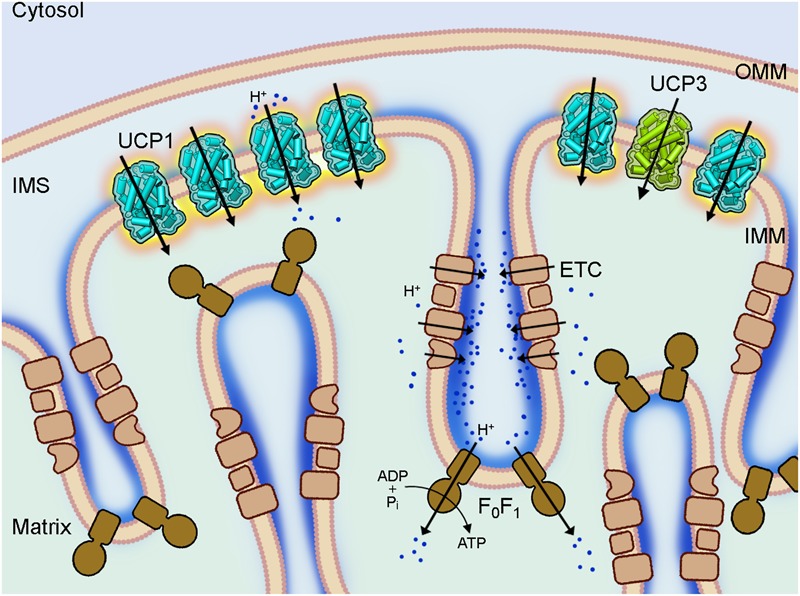
Coupling and uncoupling in mitochondria of brown adipose tissue. A section of a brown fat mitochondrion with outer mitochondrial membrane (OMM), intermembrane space (IMS) and cristae of the inner mitochondrial membrane (IMM) is shown. The complexes of the electron transport chain (ETC, beige) shuttle protons (dark blue) across the IMM and create a proton gradient, which conserved energy drives the ATP synthesis by the ATP synthase (F_0_F_1_, brown) in the cristae. Uncoupling protein 1 (UCP1, light blue), which is largely present in brown fat mitochondria IMM short-circuits the coupling of ECT and F_0_F_1_ by mediating a proton leak and dissipating the conserved energy as heat. The homologous UCP3 (green) with a similar proton transport activity is also present in the IMM but at much lower amount and its biological function is still unknown.

Uncoupling protein 1 (UCP1, previously called thermogenin) is a main player in the energy dissipation process in BAT (Figure 1) and BrAT (Shabalina et al., 2013). It is the most investigated member of the uncoupling protein subfamily that belongs to the mitochondrial anion transporters superfamily (SLC25, for review; Palmieri, 2013). UCP subfamily formally comprises five members (UCP1-UCP5). UCP1 was discovered in the mid-1970s in the mitochondria of hamster, rat and guinea pig BAT by several groups (for reviews Nicholls, 2017; Ricquier, 2017). It is regarded by several groups as the only “true” uncoupling protein (Nedergaard et al., 1999; Ricquier, 2011; Nicholls, 2017) because it dissipates the proton gradient over the inner mitochondrial membrane (IMM) to produce a remarkable amount of heat if upregulated in mammals under cold acclimation conditions.

The function of another member of the UCP family that was found in BAT, UCP3, has also been increasingly associated with obesity and diabetes (Harper et al., 2002; Costford et al., 2008; Holloway et al., 2009). However, results from different research groups are often contradicting and largely affected by the usage of non-specific antibodies. The underlying mechanisms of UCP3 functioning are obscure. UCP3 was long time handled as a twin of UCP2 due to their very high homology and history of their discovery. Meanwhile, it has become clear that their biological and transport functions differ considerably. The most intriguing issue is that UCP3, which is preferentially investigated in skeletal muscle and heart, is much more abundant in BAT [Hilse et al., 2016b; see section below]. However, UCP3 role in BAT and BrAT remains enigmatic due to the high abundance of UCP1.

In this review, we outline current knowledge on UCP3 expression and transport functions. We discuss its putative involvement in BAT functions, its interplay with UCP1 and its role in other tissues. Because several excellent reviews about different aspects of UCP1 physiology have appeared in recent years (Betz and Enerback, 2018; Gaudry et al., 2018; Jezek et al., 2018; Chouchani et al., 2019), we primarily concentrate on controversial issues in UCP1 research. We especially focus on protein expression studies and functional investigations of reconstituted UCPs.

## UCP3 Is a Member of the Uncoupling Protein Subfamily

The ways how members of the mitochondrial UCP subfamily were discovered are strikingly different. UCP1 was identified due to its high protein amount in BAT, which was visible on a Coomassie stained SDS gel loaded with mitochondrial protein obtained from BAT (Ricquier and Kader, 1976; Heaton et al., 1978). UCP3 (similar to UCP2, UCP4, and UCP5) was identified through screening cDNA libraries for candidates with homology to UCP1 (Boss et al., 1997; Fleury et al., 1997; Sanchis et al., 1998; Mao et al., 1999; Haguenauer et al., 2005).

The debates, whether UCP3, alongside with other “minor” proteins, truly belongs to the uncoupling family started shortly after they were discovered (Nedergaard and Cannon, 2003). This issue remains unsettled until now (Nicholls, 2017). The main reason for that is the discrepancy between UCP3’s ability to transport protons (Macher et al., 2018) and its low expression levels (compared to UCP1, see section below) to perform essential uncoupling.

## Expression Pattern of UCP3 – New Hints for Its Putative Function(S)

### Expression of UCP3 at mRNA Level

UCP3 was first identified by screening a human skeletal muscle cDNA library. These initial studies revealed different patterns of UCP3 mRNA expression in human and rat (Boss et al., 1997; Gong et al., 1997; Vidal-Puig et al., 1997). In rats, UCP3 mRNA was mostly present in BAT, followed by muscles with glycolytic (tensor fascia latae, tibialis anterior), mixed (gastrocnemius) and slow-twitch oxidative (soleus muscle) metabolism. UCP3 mRNA at much lower levels was also described in rat heart, lung and WAT (Alan et al., 2009). In humans, UCP3 mRNA was reported primarily in skeletal muscle and in trace quantities in the heart (Boss et al., 1997; Gong et al., 1997; Vidal-Puig et al., 1997). Differences in UCP3 mRNA distribution patterns were also detected between mouse and rat (Alan et al., 2009).

### Pitfalls in the Investigation of UCP Protein Expression

#### Discrepancy Between UCP3 Gene and Protein Expression Levels

Several unique features of UCP3, and other UCPs, complicate analysis of their expression patterns. These features are most evident for UCP2, which is regulated on multiple levels: transcriptional, translational and posttranslational (Donadelli et al., 2014). An upstream open-reading frame (uORF) regulates translation of UCP2 and is only overcome in the presence of glutamine (Hurtaud et al., 2006, 2007). This leads to a strong discrepancy between protein and mRNA expression (Pecqueur et al., 2001; Rupprecht et al., 2012, 2014), making evaluation of protein levels very important for understanding of protein’s function. UCP3 and UCP5 also possess this type of uORF (Pecqueur et al., 2001). Although the discrepancy between UCP3 and *ucp3* expression levels is less obvious (Hilse et al., 2016b), it should be considered. UCP5 expression seems to be below the detection level of western blot sensitivity. Furthermore, the ratio of UCP5 mRNA to the housekeeping gene GAPDH is very low compared to UCP2/GAPDH or UCP4/GAPDH (Rupprecht et al., 2014; Smorodchenko et al., 2017).

#### Unusually Short Lifetime of UCP3

The half-life of most mitochondrial inner-membrane proteins is approximately 12 days in liver mitochondria (Brunner and Neupert, 1968). UCP3 and UCP2 share an unusually short half-life of approximately 30 min (Rousset et al., 2007; Azzu et al., 2010). In contrast to UCP1, which has a half-life of 30 h (Puigserver et al., 1992), both UCP3 and UCP2 are quickly degraded by the cytosolic proteasome (Azzu and Brand, 2010). This feature allows a very rapid adjustment of protein levels. Therefore, evaluation of data based only on RNA expression should be assessed with caution.

#### Poor Specificity of Commercial Antibodies Against Uncoupling Proteins

**FIGURE 2:**
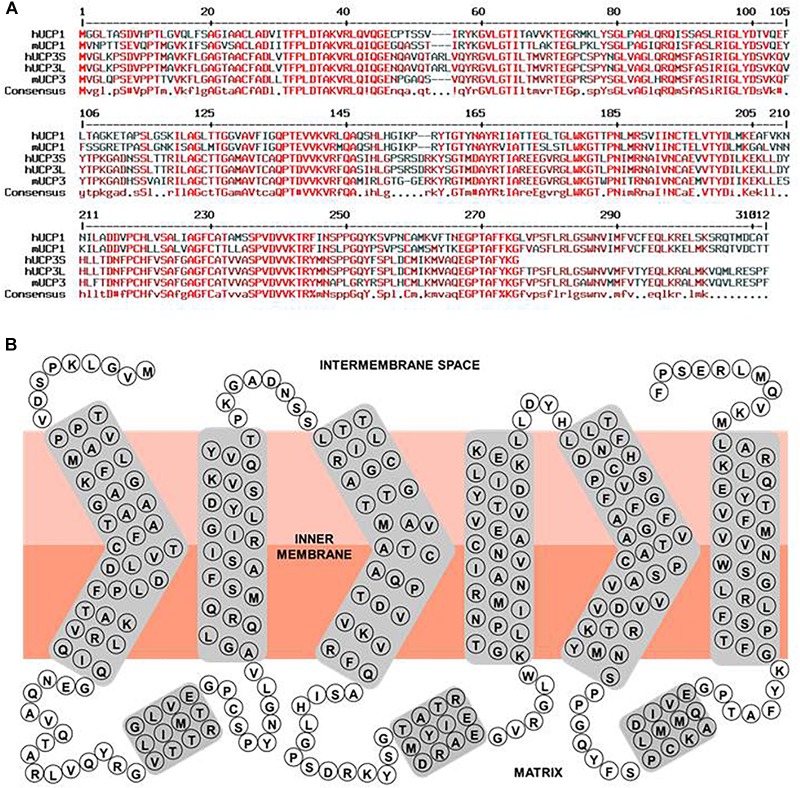
Human UCP3 primary sequence characteristics. **(A)** Multiple sequence alignment of hUCP1, hUCP3S, hUCP3L, mUCP1, and mUCP3. Amino acid sequences of human UCP1 (NP_068605.1), mouse UCP1 (NP_033489.1), human UCP3 short isoform (NP_073714.1), human UCP3 long isoform, and mouse UCP3 (NP_033490.1) were compared with respect to homology using “Multiple sequence alignment with hierarchical clustering” (Corpet, 1988). Red, dark red and black colored residues indicate homologous, similar and different residues between the proteins, respectively. **(B)** Simple scheme of the structure of the human UCP3 long isoform based on its homology to ANT and ANT crystallographic structure (Pebay-Peyroula et al., 2003).

A serious issue contributing to divergent results regarding expression patterns of uncoupling proteins in general, and UCP3 in particular, is high homology within the UCP subfamily (Figure 2A and Table 1). Moreover, homology between UCPs and other mitochondrial carriers is approximately 20% and molecular weights vary between only 30 and 36 kDa for most carriers (Table 1). This considerably hampers the design and evaluation of specific antibodies.

**Table 1:** Homology of human mitochondrial transporters.

		Homology (%)								
Protein name	MW (kDa)	UCP1	UCP2	UCP3	UCP4	UCP5	UCP6	DIC	OGC	ANT1
UCP1 Slc25a7	33.0	100	59	59	32	34	35	34	33	23
UCP2 Slc25a8	33.2	59	100	72	34	38	38	37	33	29
UCP3 Slc25a9	34.2	59	72	100	35	37	38	37	34	26
UCP4 Slc25a27	36.1	32	34	35	100	40	39	29	31	23
UCP5 (BMCP1) Slc25a14	40.1	34	38	37	40	100	80	30	38	30
UCP6 (KMCP1) Slc25a30	32.5	35	38	38	39	80	100	31	35	28
DIC Slc25a10	32.1	34	37	37	29	30	31	100	38	28
OGC Slc25a11	34.1	33	33	34	31	38	35	38	100	25
ANT1 Slc25a4	33.1	23	29	26	23	30	28	28	25	100
CIC Slc25a1	34.0	25	27	26	20	24	24	27	26	23
PiC Slc25a3	40.1	26	24	26	26	24	21	26	24	23
CAC Slc25a20	32.9	28	28	28	26	25	26	23	24	24
GC1 Slc25a22	34.5	26	25	25	21	27	26	24	24	24
AGC1 (Aralar) Slc25a12	74.8	29	27	27	25	28	28	26	29	30
APC2 Slc25a23	52.4	26	31	30	22	28	31	27	26	28

Full-length proteins are rarely used as immunogen because of high levels of homologous sequences between mitochondrial membrane proteins and difficulties with production of pure and correctly folded protein in sufficient amounts. Thus, antibodies are typically produced against a specific peptide from the target protein (Smorodchenko et al., 2009; Hilse et al., 2016b). This requires selection of a sequence that (i) shows the lowest homology to other proteins, (ii) has low hydrophobicity and is, therefore, not located in the membrane. Most antibodies against UCPs that are commercially available and/or used in studies are polyclonal. The documented antibody specificity to the target peptide is not sufficient for its proper evaluation. As a positive control, use of the full recombinant protein or tissue/cells with known prominent UCP3 expression (see section below) is crucial. Importantly, additional validation is required showing that the antibody does not detect other proteins. Cells or tissues from corresponding knockout (KO) mice or cellular knockdown models are the best choice for negative controls. Additionally, glutamine deprivation decreases UCP2 protein level and can be used as a negative control for antibody evaluation (Zimmermann et al., 2017; Rupprecht et al., 2019). Unfortunately, no such controls have been identified for UCP3.

The Ricquier group demonstrated poor specificity of commercially available antibodies in 2001 (Pecqueur et al., 2001). Unfortunately, antibody specificity and evaluation prior to use have not improved during the subsequent years, largely contributing to the confusion surrounding expression patterns and functions of UCP, particularly UCP3.

### Expression Pattern of UCP3 at Protein Level

The general consensus is that UCP3 protein expression is limited to skeletal muscles (SkM), heart, BAT and BrAT. Increased levels of UCP3 observed in WAT of mice housed at room temperature (Shabalina et al., 2013; Hilse et al., 2016b) are thought to originate from BrAT. Relative UCP3 abundance between tissues seems to depend strongly on the investigated species. UCP3 quantification in mouse tissues using recombinant protein revealed that BAT contains eight times more UCP3 than any SkM under physiological conditions (Hilse et al., 2016b).

UCP3 levels are much lower than those of UCP1 but are comparable to other mitochondrial carriers. Except for adenine nucleotide translocator (ANT, AAC) and UCP1, all members of the SLC25 family are present at concentrations less than 20 pmol/(mg of total protein) (Palmieri, 2013). Quantitative analysis based on western blot using recombinant protein for calibration revealed that UCP3 is present at 15.0, 1.7, 1.1, and 2.7 pmol/mg protein in BAT, gastrocnemius muscle, scapular muscle and heart, respectively (Hilse et al., 2016b). At 15.0 pmol/mg protein, maximal levels of UCP2 were detected in stimulated T-cells (Rupprecht et al., 2012). In contrast, UCP1 is present in BAT of mice not adapted to cold at nearly 4 nmol/mg protein, being 400-fold more abundant than UCP3 (Hilse et al., 2016b). Consequently, only UCP1 can be purified from tissue, whereas UCP3 and other mitochondrial carriers are typically overexpressed in heterologous systems, such as *E. coli*, yeast, mammalian cells, insects, etc. for further investigation in biomimetic systems (Hirschberg et al., 2011).

### UCP3 Abundance and Fatty Acid Oxidation – A New Concept

Analysis of UCP3 protein expression pattern in mice (BAT > > heart > muscles) implies that its presence correlates with a definite type of cellular metabolism – FA β-oxidation (FAO). BAT mitochondria have a high capacity for utilizing free long-chain FA as a substrate for β-oxidation (Cannon and Nedergaard, 2004). Although both, FA and glucose can be used for immediate energetic supply, FAs from internal stores are preferentially used (Bargut et al., 2016). In contrast, SkM use glucose/glycogen in a resting state and during short activity, whereas they break down lipids and even proteins during prolonged exercise (Egan and Zierath, 2013). Indeed, increased UCP3 mRNA in response to prolonged muscular contraction has been reported by several groups (Cortright et al., 1999; Zhou et al., 2000). At protein level, UCP3 was also proposed to be positively affected by exercise (Zhou et al., 2000; Ljubicic et al., 2004).

The correlation between UCP3 levels and degree of FAO was investigated in murine heart (Hilse et al., 2018). This model has the advantage that cardiomyocyte metabolism gradually changing at different stages of maturity from predominantly glycolysis in embryonic heart to FAO in adult hearts (Lopaschuk and Jaswal, 2010). Notably, both UCP3 abundance and dominance of FAO reach a maximum in 2 months old mice and decline again in old mice that increasingly utilize carbohydrates.

Increases in *ucp3* gene expression, which are induced by augmented lipid levels in blood plasma, e.g., during fasting/starvation, high fat diet (HFD), cold exposure (Matsuda et al., 1997; Millet et al., 1997; Cadenas et al., 1999) and after direct FA supply (Weigle et al., 1998), further support the correlation between FAO and UCP3 abundance. At the protein level, UCP3 was characterized under HFD conditions, revealing a 2.5-fold increase in UCP3 in the heart of wild type (wt) mice fed with HFD (Boudina et al., 2012). Increased abundance of skeletal muscle UCP3 protein was also shown in mice under caloric restriction (Bevilacqua et al., 2005), implying again that UCP3 is sensitive to increased lipid levels in the blood independently of lipid origin (exogenous or endogenous).

**FIGURE 3:**
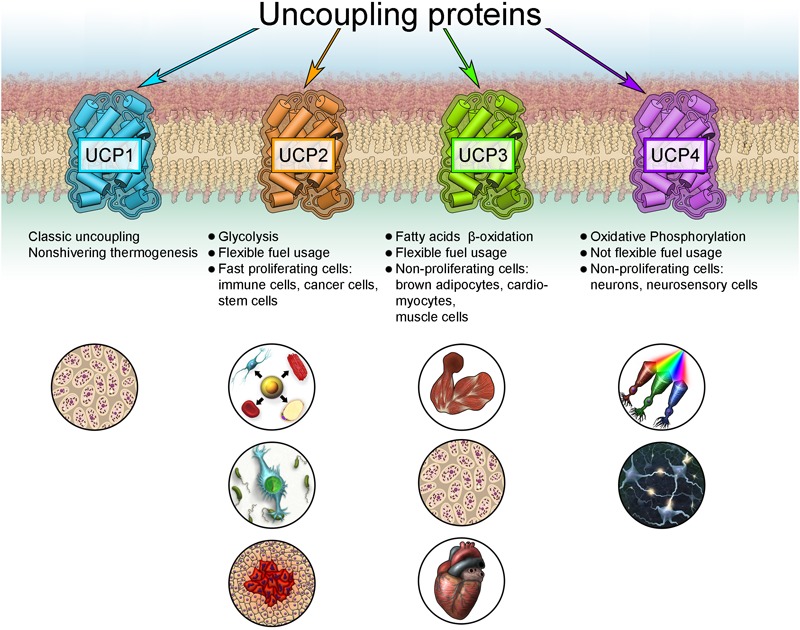
New concept for the expression of uncoupling proteins.

FAO-dependent UCP3 expression fits into the new concept of cell metabolism-specific UCP expression, originally proposed for UCP2 and UCP4 (Rupprecht et al., 2014). UCP2 is primarily identified in cells with high synthetic and proliferative activity, such as pluripotent stem, cancer and immune cells, including microglia in the brain (Figure 3). These cells support their metabolism mainly by aerobic glycolysis. This cell and tissue distribution is further supported by several studies using validated UCP2 antibodies (Pecqueur et al., 2001, 2009; Yu et al., 2013). In contrast, UCP4 protein is abundant in highly active cells with low proliferation potential that rely on a stable supply of glucose, such as neurons and neurosensory cells (Mao et al., 1999; Liu et al., 2006; Smorodchenko et al., 2009, 2011, 2017; Rupprecht et al., 2014).

Because the preferred metabolism of most cells and tissues depends on many physiological conditions that may change under pathological conditions, this concept may partly explain controversial reports concerning UCP expression, particularly for UCP2 in brain, heart, etc. During inflammation or allergic reaction, activated lymphocytes or mast cells massively invade the affected organ, potentially yielding false positive results for expression of UCP2 in inflamed organs (Smorodchenko et al., 2017). It was shown that UCP2 is highly abundant in embryonic heart and during early stages of heart development, coinciding with the glycolytic type of cardiomyocyte metabolism (Hilse et al., 2018). The ubiquitous presence of UCP2 mRNA may be required for rapid protein production to adapt to changing metabolic conditions, for instance, facilitating rapid proliferation in activated immune and cancer cells (Rupprecht et al., 2012, 2014).

## Transport Function of UCP3 and Its Regulation

### Short Description of UCP3 Structure

To date, only the 3D crystallographic structure of adenine nucleotide translocase (ANT) has been solved among all mitochondrial carriers (Pebay-Peyroula et al., 2003). The NMR structure of UCP2 obtained in the presence of dodecylphosphocholine (Berardi et al., 2011) is under discussion with regard to its physiological relevance (Zoonens et al., 2013). The structures of other mitochondrial carriers have been modeled based on their homology to the ANT (Table 1).

UCP3 is a typical mitochondrial carrier with a 312 amino acid peptide chain in mouse, arranged in six transmembrane domains that are connected by three long matrix loops and two shorter intermembrane (IMS) space loops (Figure 2B). N- and C-termini are located in the IMS. The protein displays a tripartite structure. Each part is composed of two transmembrane helices joined by a matrix loop comprising approximately 100 amino acids with the highly conserved mitochondrial carrier motif PX[D/E]XX[K/R]. UCP3 has 59% homology to UCP1 (Table 1). Two splice variants of UCP3 have been discovered in human (Solanes et al., 1997). The long form has a length of 312 amino acids (aa) and a molecular weight (MW) of 34.2 kDa. The short isoform is missing sixth helices. It has a length of 275 amino acids and MW = 29.8 kDa. The relevance of splice variants is still uncertain.

### Fatty Acid-Activated Proton Transport Is a Function of UCP3 Verified in Biomimetic Systems

Whereas measurements of the enhanced proton leak led directly to the discovery of UCP1 (Nicholls, 2017), the conclusion about UCP3 proton-transporting function was first derived from measurements of mitochondrial potential, Φ_m_, in yeast overexpressing UCP3 (Gong et al., 1997). This result was questioned in studies employing KO mice and isolated mitochondria (Thompson and Kim, 2004; Lombardi et al., 2010; Nabben et al., 2011b; Georgiadi et al., 2012; Lee et al., 2013).

Direct proof for UCP3’s ability to transport protons in the presence of FA was obtained in biomimetic systems – liposomes and planar bilayer membranes (Zackova et al., 2003; Macher et al., 2018). H^+^ transport rate of reconstituted recombinant UCP3 (2.6/s; Macher et al., 2018) was similar to UCP2 (4.5/s; Beck et al., 2007) but was fivefold lower than that of UCP1 (13.5/s; Urbankova et al., 2003). The difference between UCP1 and UCP3 would only be relevant at high protein concentrations, such during cold acclimation. Proton transport rates estimated for UCP1 using other models range from 1 to 700/s (reviewed in Hirschberg et al., 2011).

It was directly shown both in isolated mitochondria (reviewed in Ricquier, 2017) and in reconstituted systems (Urbankova et al., 2003; Klingenberg, 2017) that UCP1-mediated proton transport is activated only in the presence of free long-chain FA. Indirect evidence is obtained from experiments with UCP3 KO mice, showing a lack of effect on basal proton conductance of isolated mouse mitochondria (Cadenas et al., 2002). In mitochondrial membranes, free FAs are produced by PLA2 from phospholipids in sufficient amounts to activate UCPs (Jezek et al., 2015). In the venous blood, free FA concentrations vary from ∼0.25–3.0 mmol/l, depending on food supply and exercise. Their levels significantly increase under pathological conditions, such as obesity and diabetes (Hamilton and Kamp, 1999) or locally. Typically, saturated FAs (myristic, palmitic, stearic) are added in micromolar concentrations to mitochondria to activate UCP1-UCP3 to avoid interference with cellular pathways or FA oxidation. Direct comparison of UCP3 activation in response to different FAs comes from experiments using biomimetic systems (Zackova et al., 2003; Macher et al., 2018). Activation efficiency increases with increased FA unsaturation and chain length in the order of palmitic < oleic < eicosatrienoic < linoleic < retinoic < arachidonic acids as shown for UCP1 and UCP2 (Zackova et al., 2003; Beck et al., 2007). The strongest activation for UCP3 was obtained in the presence of arachidonic acid (Macher et al., 2018).

Several other molecules have been proposed as direct activators of UCP1 and UCP3 (for review Crichton et al., 2017). However, reports that coenzyme Q10, superoxide and H_2_O_2_ activate these proteins in the absence of free FAs have not been confirmed (Jaburek and Garlid, 2003; Esteves et al., 2004; Lombardi et al., 2008).

The lipid oxidation products hydroxynonenal (HNE) and oxononenal (ONE) do not activate UCPs directly but strongly enhance the proton transport (Malingriaux et al., 2013; Jovanovic et al., 2015) if directly added to the UCP reconstituted in bilayer membrane in the presence of FA (this situation corresponds to local production of reactive aldehydes under oxidative stress). Interestingly, this effect was only recorded if membranes contained phosphatidylethanolamine (PE). The formation of HNE-PE and ONE-PE adducts, but not direct binding to the positively charged amino acids of the protein, was responsible for this effect (Jovanovic et al., 2015).

Interaction of UCP3 with HNE was investigated in SkM and heart in connection with its putative ROS-regulating role (Echtay et al., 2003; Aguirre and Cadenas, 2010; Nabben et al., 2011b; Lopez-Bernardo et al., 2015). No consensus has been achieved on whether HNE directly influences UCP3 and UCP2 activity. Our own results demonstrated that HNE added to UCP2-expressing neuroblastoma cells leads to decreased mitochondrial potential. The latter, however, does not depend on the presence of UCP2 (Zimmermann et al., 2017). ROS regulation can also occur indirectly via reversible glutathionylation on cysteine residues in UCP3 (Mailloux et al., 2011).

Transmembrane potential, Φ_m_ is an important factor for UCP activation. Protein activity in the presence of FA increases non-linearly at physiologically relevant potentials 130–220 mV (Rupprecht et al., 2010), implying that Φ_m_ is crucial for regulation of UCP-mediated proton leak at constant free FA levels. Recent research revealed that UCPs are very sensitive to alteration of physico-chemical membrane parameters, such as surface potential and membrane fluidity (Malingriaux et al., 2013; Jovanovic et al., 2015). Interestingly, alteration of dipole potential in the presence of phloretin (Pohl, 2005) did not affect the proton activity of UCP1 (Jovanovic et al., 2015).

Conductance of membranes reconstituted with FA and UCP strongly depends on pH. The pK_a_ of the FA carboxyl group depends on FA structure and shifts from approximately 4.75 to 6.5–7.5 upon FA incorporation in the membrane (Hamilton, 1998; Pohl et al., 2008; Pashkovskaya et al., 2018). Maximum UCP activity was measured at pH values that coincide with pK_a_ values of the activating FA (Rupprecht et al., 2010).

### Putative Mechanisms of UCP3-Mediated Proton Transport

It is still unclear how FAs activate the protonophoric activity of UCPs. Although the discussion is mainly based on experiments performed with UCP1, the conclusions are typically extended to UCP3.

**FIGURE 4:**
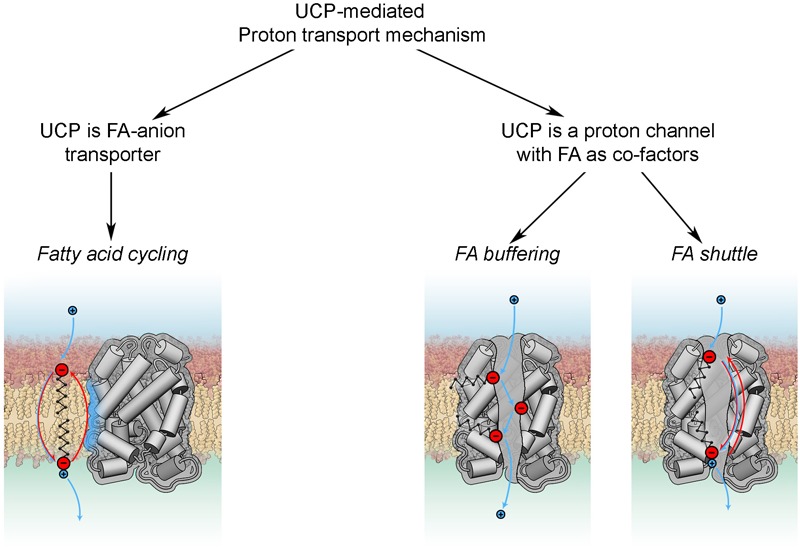
Proton transport mechanisms.

Basically, all existing models can be divided in two groups according to the roles attributed to FA and UCP (Figure 4). The *fatty acid cycling* hypothesis, introduced by Garlid et al. (1998) and Skulachev (1991), regards the protein as an anion transporter. Proton transport is performed by FA in its neutral form through a so-called “flip-flop” mechanism (Kamp and Hamilton, 1992). This concept is in agreement with classification of UCPs as mitochondrial anion transporters. Classical support for this hypothesis comes from experiments with sulfonated FA homologs, which cannot be protonated and therefore do not activate UCP1 (Garlid et al., 1996) or UCP2 (Berardi and Chou, 2014). The dependence of H^+^ transport rate on FA saturation, FA chain length (Beck et al., 2007) and fluidity of the membrane (Jovanovic et al., 2015) indicates that FA^-^ transport likely occurs at the protein-lipid interface.

In the second group of hypotheses (Figure 4) UCPs (mainly discussed for UCP1) are regarded as proton transporters that function in complex with FAs. The *FA buffering* model (for review Klingenberg, 2017) postulates that FAs bind in the protein cavity that makes their carboxyl groups available for protons to translocate from the IMS to the mitochondrial matrix through the channel (Klingenberg and Huang, 1999; Klingenberg, 2010). The recently proposed *FA shuttle* model may be regarded as a modification. It states that FA anion binds inside the pore from the cytosolic side of UCP1 and transfers protons by shuttling from the cytosolic to matrix side (for review Bertholet and Kirichok, 2017). This model is based on the inability of UCP1 to bind FAs on the matrix side, which was demonstrated in patch-clamp experiments on mitoplasts (Fedorenko et al., 2012). This contradicts the well-established view that FA binding to protein occurs from the matrix side. A further shortcoming of this model is the assumption that hydrophobic interaction of FA with the protein is much stronger than with membrane lipids. Several experiments, such as the addition of alkylsulfonates and establishment of a FA gradient, cannot be unambiguously interpreted in favor of the FA shuttle model, as comprehensively discussed in Jezek et al. (2018). Both models, FA buffering and FA shuttle, fail to explain the dependency of UCP proton transport rates on FA structure (Zackova et al., 2003; Beck et al., 2007) and membrane fluidity (Beck et al., 2007).

### Comparison of UCP1 and UCP3 Inhibition by Purine Nucleotides (PN)

It is generally accepted that the protonophoric activity of UCP1 and UCP3 is inhibited by PN. The mechanism of UCP1 inhibition was proposed by Garlid‘s and Klingenberg‘s groups 20 years ago (Modriansky et al., 1997; Klingenberg, 2010). Three arginine residues, R84^∗^, R183^∗^, and R277^∗^, located in the UCP1 funnel, were postulated to be crucial for the interaction with the PN phosphate groups and protein inhibition. According to this model, PN binding to UCP1 occurs in three steps: (1) the β-phosphate of PN binds to R183^∗^ to form the loose conformation; (2) either the γ-phosphate (ATP, GTP) or β-phosphate (ADP, GDP) binds to R84^∗^ to generate a tight conformation, and finally, (3) α-phosphate binds to R277^∗^, triggering protein conformational change and inhibition. Thus, binding strengths and inhibition of di- and triphosphates were supposed to be identical, while monophosphates are unable to bind.

Recently, we compared binding forces between different nucleotides and UCP1-UCP3 at the single molecule level using a combination of recognition imaging and force spectroscopy (Koehler et al., 2017). We revealed that bond lifetimes of both mUCP3-PN and mUCP1-PN interactions decreased in proportion to the degree of PN phosphorylation. These results are in agreement with the overall strength of PN inhibition that decreases in the order ATP > ADP > AMP in electrophysiological experiments (Macher et al., 2018).

For the first time, the combination of recognition and force modes of atomic force microscopy (AFM) (Koehler et al., 2017) have allowed estimation of the depth of the nucleotide binding side from the membrane surface. It was shown to be 1.27 nm (Zhu et al., 2013). Nucleotides were able to bind to UCP1 from both the intermembrane and matrix sides. However, only binding from the intermembrane side led to protein inhibition. This finding explains why UCPs may still exhibit protonophoric activity, even at high PN concentrations permanently present in the mitochondria.

**FIGURE 5:**
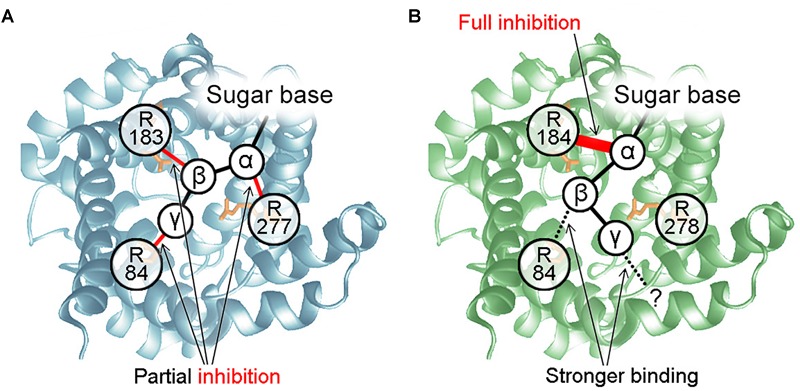
Mechanisms of UCP-PN interaction and inhibition. **(A)** PN inhibition mechanism for UCP1. The α-, β-, and γ-phosphate of PNs bind to R277, R183, and R84, respectively. R84 does not interact with the β-phosphate of diphosphate-PNs. The three P-R bonds additively contribute to maximum inhibition but interact independently. None of them is essential for inhibition or PN binding. **(B)** Mechanism of UCP3 inhibition by PNs. R184 and R84 bind to the α-and β-phosphate of PNs. Interaction between R184 and the PN α-phosphate is essential for protein inhibition and may induce a conformational change. Interaction of R84 with the β-phosphate increases binding strength. Instead of R278, another residue is proposed to be a part of the UCP3 PN-binding-pocket, which binds to the γ-phosphate of PN.

We demonstrated that UCP3 reconstituted in bilayer membranes is completely inhibited by all PNs, regardless of phosphorylation, but IC50 increases as phosphorylation decreases. This contradicts the previous assumption that diphosphate-PNs are the most potent inhibitors of UCP3 (Echtay et al., 1999; Zackova et al., 2003). However, observations of UCP3 helical content using circular dichroism (CD) (Ivanova et al., 2010) support that triphosphate-PNs exert the strongest effect on UCP1/UCP3 conformation. Experiments with mutated arginines allowed us to propose a mechanism for UCP3 inhibition (Figure 5). It postulates that the interaction between R183 and α-phosphate of PN is essential for UCP3 inhibition and, by itself, causes full inhibition. The IC50 of inhibition is further decreased by bond formation between arginines and PN β- and γ-phosphates. In contrast to its important role in UCP1 inhibition, R277 is not a part of the UCP3-PN binding-pocket. R84 interacts with the β-phosphate of PN, while the residue that binds the γ-phosphate remains unknown (Macher et al., 2018).

We also re-evaluated the mechanism of inhibition for UCP1. We first revealed that interaction between the α-phosphate of AMP and arginine is sufficient for binding and partial inhibition of UCP1. We further proposed that each arginine-phosphate interaction contributes equally and additively to maximum inhibition, resulting in complete inhibition in the case of three interactions (α-, β-, and γ- phosphates, Figure 5). In contrast, it was shown earlier, that site-directed mutagenesis of R277 completely abolishes GDP inhibition of proton transport by UCP1 (Murdza-Inglis et al., 1994).

### Other Inhibitors of UCP3

It is very likely that molecules capable of binding to the above mentioned arginines, might be putative inhibitors of UCP3. For example, genipin was described as a specific inhibitor of UCP2 (Zhang et al., 2006). Recently, we have shown that genipin also decreases the protonophoric activity of UCP1 and UCP3 (Kreiter et al., 2018). Several chromane derivatives inhibit UCP1 and UCP2 (Rial et al., 2011), but have not been tested on UCP3. Organic phosphate, P_i_, decreases the activity of UCP1, UCP2, and UCP3 reconstituted in planar bilayer membranes up to 60% (Macher et al., 2018). Inhibition by P_i_ was independent of the presence of arginine residues in the PN-binding pocket, implying a different molecular mechanism from that of PNs.

## Putative Biological Functions of UCP3

Meanwhile UCP3 was proposed to be involved in all relevant pathophysiological states. Several studies have identified polymorphism of UCP genes that are associated with fat metabolism, obesity and diabetes (for review Jia et al., 2009).

### Conclusions From UCP3 KO Mice and Cells Overexpressing UCP3

With the creation of the UCP1 KO mouse (Enerback et al., 1997), the function of UCP1 as a molecular basis of non-shivering thermogenesis was verified. UCP1 KO mice were shown to rely on shivering for thermoregulation (Golozoubova et al., 2001). In contrast, UCP3 KO mice did not contribute to uncovering the precise function of UCP3 (Gong et al., 2000; Vidal-Puig et al., 2000). UCP3 KO mice showed no obvious phenotype or changes in general behavior compared to their wt littermates under physiological conditions. Analysis of isolated mitochondria revealed a more uncoupled state with simultaneous increase in ROS production. Despite these changes, UCP3’s contribution to weight regulation, FA oxidation and non-shivering thermogenesis was not substantial. Even under challenging conditions as high-fat diet, fasting, stress, cold exposure and thyroid hormone treatment UCP3 KO mice were indistinguishable from wt littermates. Double KO mouse (UCP1/UCP3 dKO) revealed the same phenotype as a single UCP1 KO mouse (Gong et al., 2000).

Investigation the physiological role of UCP3 in skeletal muscle by challenging UCP3 KO mice did not lead to conclusive results. A protective role of UCP3 during lipotoxicity was not confirmed (Nabben et al., 2011a), but was later found to be relevant in the heart (Nabben et al., 2014). UCP3 was not required during fasting in skeletal muscle, and FA anion export occurred independently of UCP3 (Seifert et al., 2008). In contrast, UCP3 was suggested to protect mitochondria against lipid-induced damage by transporting FA anions out of the mitochondria based on experiments involving short and long-term high-fat diet (Schrauwen et al., 2002; Costford et al., 2008).

One possible reason for the failure to detect a UCP3 KO phenotype could be the lack of a relevant trigger, as represented by cold adaptation in UCP1 KO mice. Second, it has to be considered that an organism with a complete protein KO has to adapt to modified circumstances, especially before birth. It is well known that essential life processes have several back-up pathways. The involvement of UCP3 in FA metabolism suggests a possible metabolic adaptation by preferential utilization of other metabolic pathways (e.g., glycolysis). The high homology between mitochondrial carriers, especially UCPs, has prompted a “take over” hypothesis (Nedergaard and Cannon, 2003). However, for UCP1, UCP2, and UCP3 this hypothesis was refuted (Hilse et al., 2016b). Instead, dependence of UCP3 expression on UCP1 presence has been observed (Hilse et al., 2016b).

Mouse overexpressing UCP3 (UCP3 Tg) in skeletal muscle was created in the same year as KO mouse (Clapham et al., 2000). The original Tg mice exhibited a 20-fold increase in UCP3 over normal levels. Subsequently, their phenotypic characteristics were attributed to high proton leak caused by the non-physiological levels of UCP3. Successive studies involved mice that overexpressed UCP3 in skeletal muscle at lower levels (2–3-fold greater than wt mice). This mice exhibited a hyperphagic and lean phenotype with a shift toward FA transport and oxidation (Bezaire et al., 2005). Overall analysis of these mice revealed decreased body weight, as well as epididymal white adipose tissue (eWAT) and BAT deposits. Increased insulin sensitivity and impaired tolerance to glucose provided further hints of changes occurring in the whole organism (Costford et al., 2006). Moreover, caloric restriction had a higher impact on muscle loss in UCP3 Tg than in wt mice (Estey et al., 2012). This finding suggests that UCP3 overexpression in muscle imitates strong exercise, as these tissues exhibited decreased markers of incomplete β-oxidation (Aguer et al., 2013), once again connecting UCP3 function to FAO.

Importantly, the ectopic and entopic overexpression of mitochondrial carriers also faces serious problems. The described changes may be caused by experimental artifacts. When ectopically overexpressed in HEK cells, the oxoglutarate carrier was shown to cause strong mitochondrial uncoupling due to incorrect assembly into the membrane (Yu et al., 2001). On the other side, if protein function is associated with a definite type of metabolism (FAO in case of UCP3), only a matching host metabolism would deliver reliable insight into UCP3 function.

### Evidences for and Against UCP3 Involvement in Thermogenesis

Because UCP1 and UCP3 are both localized in BAT and transport protons, the first idea is that the thermogenic function of UCP1 may be taken over by its “sister” protein. Due to the similarity of their proton transport rates (Urbankova et al., 2003; Macher et al., 2018), only strongly upregulated UCP3 can substitute for UCP1 under cold acclimation conditions. However, the abundance of UCP1 increases sevenfold (Kalinovich et al., 2017), whereas UCP3 only triples its levels (Hilse et al., 2016b). Since it is nearly 400 times less abundant than UCP1, UCP3 cannot play a thermogenic role. As mentioned previously, KO of UCP3 and UCP1/UCP3 dKO did not support a thermogenic function of UCP3 (Gong et al., 2000; Vidal-Puig et al., 2000). Interestingly, the abundance of UCP3 decreases if UCP1 is knocked out (Hilse et al., 2016b), indicating a direct correlation between UCP3 and UCP1 expression. UCP3 expression in BAT, in contrast to heart and muscles, can also be induced by cold exposure, similar to UCP1. However, although UCP3 levels tripled (Hilse et al., 2016b), its levels still remained very low compared to UCP1 (128.4 ng/mg of total cellular protein) in BAT of non-cold-acclimated mice (Rupprecht et al., 2012). Notably, UCP1^-/-^ mice revealed a decreased expression of UCP3 in BAT, which was even further decreased in response to cold exposure. In contrast, UCP3 expression in heart and skeletal tissues was unaffected by cold exposure. These observations give an important hint that specific (non-protonophoric) UCP3 transport function is only required in full functioning and activated BAT. Furthermore, a connection to the previously elucidated UCP1-independent thermogenic process (for review Kazak et al., 2015) can be denied.

Recently it was reported that both UCP1 and UCP3 are important for mammalian thermoregulation (Riley et al., 2016). Riley et al. demonstrated that noradrenaline-induced hyperthermia relays on UCP1 presence, whereas lipopolysaccharide thermogenesis requires skeletal muscle UCP3 using UCP3 KO, UCP1 KO, and UCP1/UCP3 dKO mice. This unexpected conclusion remains to be verified.

### Putative Involvement of UCP3 in ROS-Regulation

Members of the uncoupling protein family have long been hypothesized to reduce oxidative stress through mild uncoupling (Brand et al., 2004; Mailloux and Harper, 2011; Jezek et al., 2018). This hypothesis is based on UCPs’ ability to transport protons, allowing for the regulation of membrane potential dependent superoxide anion generation from the electron transport chain (mild uncoupling, Skulachev, 1998). The presence of uncoupling proteins, such as UCP4, in the inner boundary membrane supports the mild uncoupling hypothesis (Klotzsch et al., 2015). In particular, UCP2 and UCP3 are often described as regulators of ROS (Krauss et al., 2005). ROS play a regulatory role in several cellular processes or lead to oxidative stress and damage. ROS are discussed in the regulation of the thermogenesis (Chouchani et al., 2017). Increased ROS was observed in both UCP3 KO (Vidal-Puig et al., 2000) and UCP3 Tg mice (Nabben et al., 2008). In the heart, UCP3 is described as cardioprotective because of its suggested anti-oxidative function (Cadenas, 2018). The hypothesis concerning the anti-oxidative function of UCP3 is controversially discussed (Shabalina and Nedergaard, 2011). The main arguments against this hypothesis are (i) the confinement of UCP3 to limited tissues and (ii) the missing correlation between UCP3 expression and ROS production. No correlation of UCP3 levels with the expression of respiratory chain complexes, the main source of ROS, was found (Hilse et al., 2018). This renders the involvement of UCP3 in ROS regulation doubtful. Of note, mild uncoupling can also be mediated by other mitochondrial carriers, e.g., ANT (Andreyev et al., 1989).

### UCP3 Involvement in Cell Metabolism

It is important to note that all proposed functions for UCP3 are highly dependent on cellular energy metabolism. Reduced cold tolerance in hamsters due to a lack of UCP3 was accompanied by reduced metabolic gene expression in BAT (Nau et al., 2007). Noteworthy, UCP3 gene expression is controlled by PPARs, fundamental nuclear receptors for cellular energy metabolism (Villarroya et al., 2007). FAs during fasting or HFD were shown to increase UCP3 mRNA. Our recent results demonstrated that UCP3 protein expression increases during food deprivation in all organs (BAT, He, SkM; Hilse et al., 2016a). The observed phenotype of UCP3 overexpressing mice indicates putative involvement of UCP3 in (FA) metabolism (Clapham et al., 2000; Thompson and Kim, 2004; Bezaire et al., 2005). Muscle cells overexpressing UCP3 exhibit a shift to FAO (Garcia-Martinez et al., 2001). A direct correlation between UCP3 and β-oxidation type of cellular metabolism was observed during heart development (Hilse et al., 2018). UCP3 expression reaches its peak with increasing density of mitochondrial cristae, appearance of lipid drops and formation of multiple connections between mitochondria and lipid drops. The role of UCP3 in FAO may be mediated by an additional transport function, e.g., FA transport into the mitochondrial matrix to support FAO. Other groups have proposed transport of FA out of the mitochondria, implying a protective role of UCP3 against triglyceride accumulation (Goglia and Skulachev, 2003; Schrauwen et al., 2006). It has to be mentioned that FAO is another source of ROS production (St-Pierre et al., 2002).

Few comparative investigations of UCP1 and UCP3 exist. One of them describes that training enhances the relationship between UCP1/UCP3 mRNA levels, which could result in higher energy efficiency, but not under a high sugar diet (de Queiroz et al., 2012). In contrast, Shabalina et al. reported that UCP3 expression in SkM increases to compensate for UCP1 KO (Shabalina et al., 2010). We recently found that UCP3 expression in BAT, which is induced by food deprivation, is diminished by knocking out UCP1. In contrast, HFD does not affect UCP3 levels although UCP1 is increased (Hilse et al., 2016a).

### Other Putative Functions

UCP3 is associated with contractile heart function (Ozcan et al., 2013; Harmancey et al., 2015; Motloch et al., 2016). However, the electrical activity was shown to be independent from UCP3 presence (Hilse et al., 2018).

Together, UCP2 and UCP3 were suggested to regulate mitochondrial calcium uptake (Trenker et al., 2007; Waldeck-Weiermair et al., 2011). These studies are controversial and have been discussed elsewhere. Direct transport was disproven (Brookes et al., 2008), but influence on calcium homeostasis by a specific metabolite transport function cannot be excluded (Bouillaud et al., 2016).

## UCP3 as a Marker for FA Oxidation

The presence of UCP3 protein in BAT, heart and muscles, as well as its changing expression pattern during organ development, cell differentiation or physiological state (as exercising muscles), clearly reflect FAO type of the cell metabolism (Hilse et al., 2016b). This observation allows to use UCP3 as a marker for cellular metabolic state. The expression ratio of UCP3 to UCP2 might be important as a diagnostic criterion for the severity of heart failure or the degree of cardiomyocyte differentiation after stem cell transplantation (Hilse et al., 2018). Furthermore, the direct correlation of UCP1 and UCP3 expression makes UCP3 suitable as a protein marker in BAT and BrAT (Shabalina et al., 2013).

## Conclusion

**FIGURE 6:**
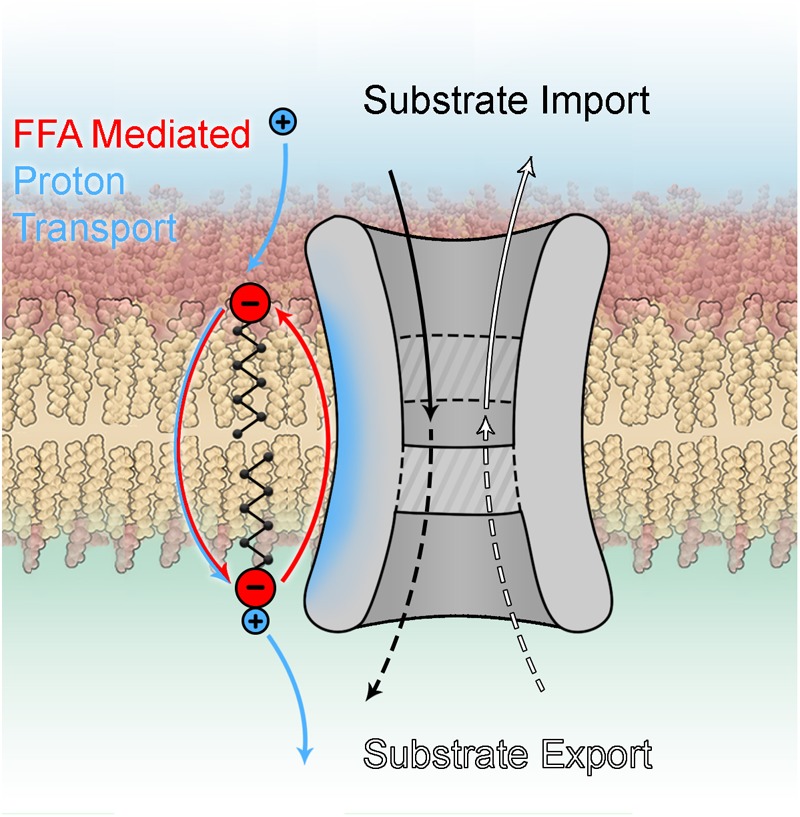
Dual function of mitochondrial carrier proteins.

(1) It has been verified that UCP3 transports protons with a rate comparable to UCP1. However, it seems very likely that its protonophoric function may be additional to another as yet unknown transport function (Figure 6) similar to other members of this family, including the oxoglutarate carrier (Yu et al., 2001), ANT (Andreyev et al., 1989), the phosphate carrier and even UCP2 (Vozza et al., 2014). This idea was already proposed by Nedergaard and Cannon (2003) but did not gain much attraction due to a lack of mechanistic insight.

(2) There are significant differences in inhibition by PN between UCP3 and UCP1. Maximum inhibition of UCP1 decreases with decreasing PN phosphorylation, while all PNs can fully inhibit UCP3. This is caused by different interaction mechanisms between PNs and arginine residues in UCP1 and UCP3. Increased free FA concentrations decrease the effect of all PNs on UCP1. In contrast, FAs affect only ATP-mediated inhibition in UCP3.
(3) P_i_ is a new inhibitor of UCP1 and UCP3 that causes partial inhibition while utilizing a mechanism distinct from that of PNs.
(4) UCP3 tissue distribution shows a clear dependence on cell metabolism, directly correlating with a preference for FAO. Expression of this protein is adaptive in nature, emerging short-term during metabolic necessity. Hence, drawing conclusions about UCP3 function based solely on mRNA data is not feasible. KO models have limited potential for the exploration of UCP3 function because mice may adapt their metabolism to the existing nutrition supply.

## Author Contributions

EP and AR contributed to the conceptualization and wrote the original draft. KH, AR, and especially GM contributed to the visualization. EP acquired funding. All authors reviewed and edited the draft.

## Conflict of Interest Statement

The authors declare that the research was conducted in the absence of any commercial or financial relationships that could be construed as a potential conflict of interest.
